# Modeling the efficacy profiles of UV-light activated corneal collagen crosslinking

**DOI:** 10.1371/journal.pone.0175002

**Published:** 2017-04-06

**Authors:** Jui-Teng Lin, Da-Chuan Cheng

**Affiliations:** 1 New Vision Inc., Taipei, Taiwan; 2 Department of Biomedical Imaging and Radiological Science, China Medical University, Taichung, Taiwan; Cedars-Sinai Medical Center, UNITED STATES

## Abstract

**Objective:**

Analysis of the crosslink time, depth and efficacy profiles of **U**V-light-activated corneal collagen crosslinking (CXL).

**Methods:**

A modeling system described by a coupled dynamic equations are numerically solved and analytic formulas are derived for the crosslinking time (T*) and crosslinking depth (z*). The z-dependence of the CXL efficacy is numerically produced to show the factors characterizing the profiles.

**Results:**

Optimal crosslink depth (z*) and maximal CXL efficacy (Ceff) have opposite trend with respective to the UV light intensity and RF concentration, where z* is a decreasing function of the riboflavin concentration (C_0_). In comparison, Ceff is an increasing function of C_0_ and the UV exposure time (for a fixed UV dose), but it is a decreasing function of the UV light intensity. CXL efficacy is a nonlinear increasing function of [C_0_/I_0_]^-0.5^ and more accurate than that of the linear theory of Bunsen Roscoe law. Depending on the UV exposure time and depth, the optimal intensity ranges from 3 to 30 mW/cm^2^ for maximal CXL efficacy. For steady state (with long exposure time), low intensity always achieves high efficacy than that of high intensity, when same dose is applied on the cornea.

**Conclusions:**

The crosslinking depth (z*) and the crosslinking time (T*) have nonlinear dependence on the UV light dose and the efficacy of corneal collagen crosslinking should be characterized by both z* and the efficacy profiles. A nonlinear scaling law is needed for more accurate protocol.

## Introduction

Photochemical kinetics of corneal collagen crosslinking (CXL) and the biomechanical properties of corneal tissue after CXL have been extensively explored in both animal and human models [1–5]. The dynamics of CXL and the safety and efficacy issues of CXL have been explored theoretically [6–13] and clinically [14, 15]. To increase the speed of CXL procedures, accelerated CXL using high UV power (9 to 45 mW/cm^2^) are also reported [16]. However, the accelerated CXL based on the linear theory of Bunsen Roscoe law (BRL) [17] to shorten the irradiation time is still a debating issue. More recent clinical studies have indicated that the efficacy based on BRL is actually lower than the non-BRL [18, 19]. Prior modeling work of Schumacher et al. The study in [6] assumes a constant riboflavin concentration during the UV light exposure time. This assumption ignoring the dynamic of the riboflavin concentration leads to major errors in the calculations of the UV light intensity profile (both spatial and temporal), the rate of photopolymerization and the increase of stiffness. The CXL efficacy is characterized by multiple factors including the concentration and diffusion profile of the RF in the stroma, the absorption constant of the photolysis products and the quantum yield of the CXL process.

The critical formulas to be developed in this study include the crosslinking time, the crosslinking rate and the efficacy defined by the conversion of monomer-polymer characterized by the following parameters: the three extinction coefficients, concentration and diffusion depth of the riboflavin solution, the UV light intensity and irradiation duration, and the corneal thickness.

This study will present, for the first time, the nonlinear law for the CXL efficacy, in contrast to the conventional Bunsen Roscoe law [17].

## Methods

### The modeling system

**Fig 1:**
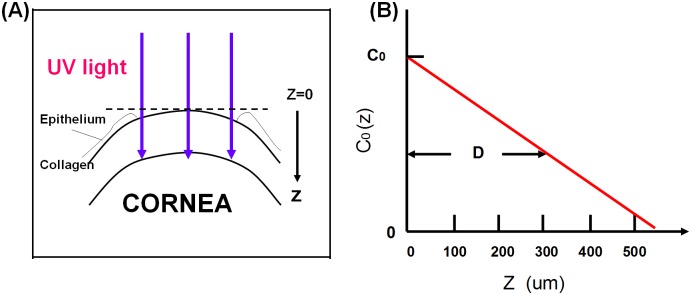
A corneal model system under UV light crosslinking. This is epi-off case. (A) The epi-off case with the stroma surface defined as z = 0. (B) The initial (at t = 0) RF concentration distribution inside the stroma given by a distribution function F(z,D), having a diffusion depth (D) [11, 12].

The corneal model system [11, 12] consists of its epithelial layer and the underlying stroma collagen as shown in Fig 1A. The UV light is normally incident to the corneal surface. The theory developed in this study can apply to both epithelium removed (epi-off) and epithelium intact (epi-on) case by slight revisions. However, we will focus on the more efficient epi-off case as shown in Fig 1B, where the riboflavin (RF) solution has an initial surface concentration (at z = 0) C_0_ and distribution in the stroma defined by C_0_(z) = C_0_ F(z, D), with F(z) = 1–0.5z/D having a diffusion depth D defined by half-width of the half-maximum, or C_0_(z = D) = 0.5 C_0_. The distribution function, F(z), is chosen to obtain analytic formulas for the roles of the diffusion depth D. One may also use a more realistic distribution based on measured data which, however, will require numerical simulations and many of the important features to be found analytically will not be available. We also assume that the UV light has a uniform intensity distribution across the stroma surface.

### The dynamic intensity

In the modeling system shown in Fig 1B, the UV light intensity *I*(*z*, *t*) and RF concentration C(z,t) in the corneal stroma (for the epi-off case) may be described by a set of coupled first-order differential equations, or by the integral equations [8–11] as follows,

$$I\text{(}z,t)=I_{0}\text{exp}\left[-2.3\int_{0}^{z}\left((\varepsilon_{1}-\varepsilon_{2})C(z\prime ,t)+\varepsilon_{2}C_{0}F(z\prime )+Q\right)dz\prime \right]$$ (1.a)

with the time-dependent RF concentration given by,

$$C\text{(}z,t)=C_{0}F(z)\text{exp}\left[-aE(z,t)\right]$$ (1.b)

$$E(z,t)=\int_{0}^{t}I(z,t\prime )dt\prime$$ (1.c)

Where *a* = 83.6*λϕε*_1_, with *ϕ* being the quantum yield and *λ* being the UV light wavelength; *ε*_1_ and *ε*_2_ are the extinction coefficients of RF and the photolysis product, respectively. I_0_ is the initial UV light surface intensity, or I(z = t = 0) = I_0_.; and C_0_ is the initial RF surface concentration assuming a distribution profile given by F(z) = 1–0.5z/D, or C(z,t = 0) = C_0_F(z), with a diffusion depth D in the stroma.

We note that the above described epi-off model may be easily applied to the epi-on case as follows. Given the epithelial thickness *d*, the intensity in Eq (1.b) is revised by redefining the corneal thickness as z’ = z + *d*, that is, extra absorption of the epithelium reduces the intensity on the stroma surface (at z = 0) by a factor of exp(-Ad), where A is the absorption coefficient of the epithelium which may be slightly different from that of stroma.

**Fig 2:**
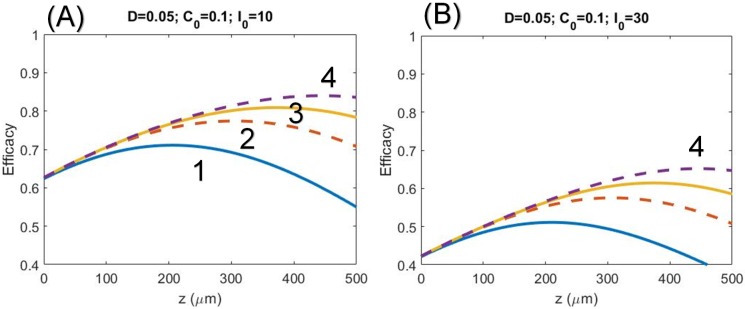
Profiles of the CXL efficacy. CXL efficacy versus corneal thickness (z) for D = 500 um, C_0_ = 0.1%, and quantum yield *ϕ* = 0.5 and for: (A) UV light intensity I_0_ = 10 mW/cm^2^ for various exposure time t = (3,5,7,10) sec, or dose of (0.03, 0.05, 0.07, 0.1) J/cm^2^ shown by curves (1, 2, 3, 4), respectively; and (B) for I_0_ = 30 mW/cm^2^ with the same dose of (A).

**Fig 3:**
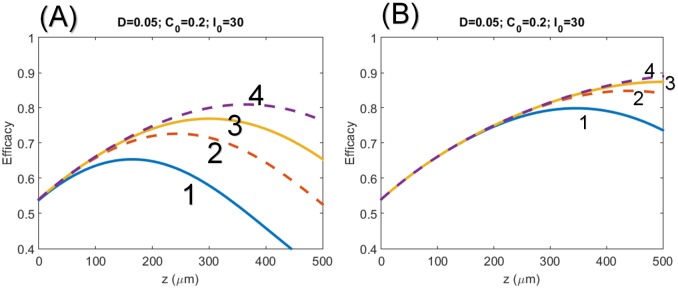
Profiles of the CXL efficacy. Same as Fig 2, but for high RF concentration and high power of C_0_ = 0.2% and I_0_ = 30 mW/cm^2.^ for: (A) transient low dose state with exposure time t = (1.0, 1.67, 2.3, 3.3) sec and (B) high dose state with t = (3, 5, 7, 10) sec.

The prior work of Schumacher et al [6] based on a non-depleted RF concentration, i.e., the assumption of *a*E = 0 in Eq (1.b), significantly overestimates the RF concentration for t>0. This assumption is also based on the continuing instillation of the RF drop during the CXL process such that the RF is always in its saturated state without depletion. That is, after the pre-treatment time, they treat the CXL as a steady process without solving the dynamics of CXL. They also use an oversimplified model to assume no absorption from the photolysis products, i.e.,*ε*_2_ = 0. Therefore, their calculated profiles, Figs 2 and 3, significantly deviate from our exact numerical profiles to be shown later.

The reported measurements [20, 21] provide the parameters *ε*_1_ = 204 (%·cm)^-1^ and Q = 13.9 (cm^-1^), whereas *ε*_2_ is not yet available in human, but is estimated to be in the range from 80 to 120 (%·cm)^-1^ by the RF depletion test [11]. The quantum yield *ϕ* will be treated as a free parameter in our calculation and have a range from 0.3 to 0.5. The following units are used: C(z,t) in weight percent (%), I(z,t) in (mW/cm^2^), *λ* in cm, Q in (cm)^-1^ and *ε*_*j*_ (for j = 1,2) in (%·cm)^-1^. As shown by Eq (1), there are three major UV absorption components in the CXL process: the absorption of the stroma tissue (Q), which is independent to the RF concentration; the absorption of the unreacted RF solution (*ε*_1_C_0_), and the photolysis product (*ε*_2_C_0_), both are proportional to the initial RF concentration C_0._ The initial UV light intensity (at t = 0) is obtained by the integration of Eq (1.a) with C(z,0) = C_0_ F(z) as follows:

$$I_{1}(z,0)=I_{0}\text{exp}(-A_{1}z)$$ (2.a)

and the steady state light intensity is derived by using *C*(*z*, *t* = ∞) = 0 in Eq (1.a),

$$I_{2}(z,\infty )=I_{0}\text{exp}(-A_{2}z)$$ (2.b)

where (for j = 1, 2)

$$A_{j}=2.3\left[Q+\varepsilon_{j}C_{0}G(z)\right]$$ (2.c)

G(z)=[1−0.25z/D], (2.d)

where A_1_ (for j = 1) is the initial state (at t = 0) absorption coefficient, independent to *ε*_2_; A_2_ (for j = 2) is the steady-state absorption coefficient, which is independent to *ε*_1_ because of the complete concentration depletion, *C*(*z*, *t* = ∞) = 0. Notably, G(z) in Eq (2.d) is the integration of F(z) over z.

### The effective dose

The effective dose (or fluence) applied to the cornea collagen (at a depth z) for a UV exposure time (t) is defined by

$$E(z,t)=\int_{0}^{t}I(z,t\prime )dt\prime$$ (3)

The UV light intensity is a dynamic function of time and z and requires a full numerical simulation of Eq (2). For comprehensive analytic formulas, various numerically fitting techniques are presented earlier [11]. In this study we will use the linear approximation of the light intensity *I*(*z*, *t*) given by the mean value of the initial (I_1_) and steady intensity (I_2_) defined by *I*(*z*, *t*) = 0.5[*I*_1_(*z*) + *I*_2_(*z*)] for t<T and *I*(*z*, *t*) = *I*_2_(*z*) + 0.5[*I*_1_(*z*)−*I*_2_(*z*)] for steady-state, or t>T, where T is the steady-state cross-linking time given by T = m/(aI_0_), with m = 12 fit numerically with the exact solution of Eq (2) [12]

Time integration of the light intensity, the effective dose (at z) Eq (3) becomes [12]

$$E(z)=E_{0}\text{exp}(-A_{2}z)-g,$$ (4)

where E_0_ = I_0_t is the surface dose (at z = 0), and g is a correction factor for the transient state given by g = 0.5(I_2_ –I_1_)(T/E_0_), T = f/(aI_0_), with the fit parameter f = 12. With *ε*_1_ = 204 (%·cm)^-1^, *ε*_2_ = 102 (%·cm)^-1^, Q = 13.9 (cm^-1^) and C_0_ = 0.1%, we obtain an approximated value of the correction factor *g* = 0.97[exp(−*A*_2_*z*)−exp(−*A*_1_*z*)], which is about 10% to 25% correction to the steady-state when E_0_ = 3.0 to 4.0 J/cm^2^ and at z = 400 um.

### The crosslinking time

Crosslinking time (T*) may be defined in various ways. Basically, it is defined when the crosslinking procedure is mostly completed, or when the RF initial concentration is mostly depleted by the UV light and the procedure reaches a steady state having a very low polymerization rate. We choose to define T* by one of our most preferred definition based on the level of RF concentration depletion as follows. T* is defined to be *C*(*z*, *t* = T*) = *C*_0_*F*(*z*)exp(−*M*), where M is the degree of the RF depletion.

Substituting Eqs (4) to (1.b), we obtain the analytic formula for the RF concentration as follows

$$C(z,t)=C_{0}F(z)\text{exp}\left[-aE_{0}\text{exp}(-A_{2}z)+agz\right].$$ (5)

Eq (5) provides us the formula for the crosslinking time defined by *C*(*z*, *t* = T*)/*C*_0_*F*(*z*)(*e*^−*N*^), which leads to the formula for the cross linking time given by

$$T^{*}(z)=T_{0}(1+ag/M)\text{exp}(A_{2}z).$$ (6.a)

Using the numerically fit expression of g, we obtain

$$T^{*}(z)=T_{0}\left[\text{exp}(A_{2}z)+1.5(1-\text{exp}(-A\prime z))\right],$$ (6.b)

where A’ = (A_1_-A_2_) and T_0_ is the surface crosslinking time given by

$$T_{0}=T^{*}(z=0)=1000N/(aI_{0})$$ (7)

for I_0_ in (mW/cm^2^), *a* = 83.6*λε*_1_*ϕ* = 0.0622*ϕ*, with *ϕ* being the quantum yield; and E_0_ = I_0_T_0_ = N/a is the surface crosslinking dose. For N = 4, T_0_ = 6.44/(I_0_*ϕ*). For example, for I_0_ = 10 mW/cm^2^, quantum yield *ϕ* = 0.5, we obtain T_0_ = 1.3 seconds, and surface crosslinking dose (I_0_T_0_) is 0.013 J/cm^2^.

### The efficacy profile

The kinetic equation of the monomer concentration [M] is given by [6, 13, 22]

$$\frac{\partial \text{[M](}z,t)}{\partial t}=-R(z,t)[M]$$ (8.a)

$$R(z,t)=\sqrt{aKC(z,t)I(z,t)}$$ (8.b)

where K is the ratio of the growth and termination rate constant of the polymer chain.

Solving for Eq (8) we obtain the degree of the monomer-polymer conversion, which is proportional to the CXL efficacy (Ceff) as follows.

$$C_{eff}=1-\frac{\left[M\right]}{{\left[M\right]}_{0}}=1-\text{exp}\left[-S(z,t\prime )\right]$$ (9.a)

$$S(z,t)=\int_{0}^{t}R(z,t\text{')}dt\text{'}$$ (9.b)

Analytic formulas have been discussed for the simplified case that the RF depletion is ignored, and for the case that absorption of UV light in the stroma (without RF) is ignored (or Q = 0) [22]. Analytic formulas including both Q and the RF depletion can be obtain by using the mean UV light intensity such that I(z,t) = 0.5 (I_1_ + I_2_) with I_j_ defined by Eq (2), as follows.

$$R(z,t)=\sqrt{KC_{0}F(z)I_{0}H(z)}\text{exp}(-0.5aE(z))$$ (10.a)

$$H(z)=0.5\left[\text{exp}(-A_{1}z)+\text{exp}(-A_{2}z)\right]$$ (10.b)

and the E(z) in Eq (10.a) may be approximated by E(z,t) = tI_0_H(z), we obtain the profile (z-dependence) of S(z,t) as follows:

$$S(z,t)=P(z,t)\sqrt{4KC_{0}F(z)/(aH(z)I_{0})}$$ (11.a)

$$P(z,t)=1-\text{exp}\left[-0.5atH(z)I_{0}\right]$$ (11.b)

Eqs (8) and (10) shows that photoinitiation rate (R) is a product of two competing factors, the RF concentration and the UV light intensity. Taking dS/dz = 0, we may find the optiomal z* for maximum S and Ceff, and z* is proportional to the dose, or exposure time for a given UV light intensity. Eq (11) also shows that the steady state efficacy (when *atHI*_*0*_ >>1), is inverse proportional to the square root of [aI_0_] which will be further demonstrated by our numerical results later. Defining a steady state by when P(z,t) < 0.133 and almost independent to time, we obtain the condition for a steady-state dose as follows: E_0_ = tI_o_ > 0.026 exp [H(z)], for E_0_ in (mJ/cm^2^).

We may solve for Eqs (9) and (11) to obtain the exposure time formula for a given CXL efficacy as follows.

$$t=\left(\frac{-2}{x}\right)\text{ln}\left[1-B\sqrt{x}\right]$$ (12.a)

$$B=-\text{ln}(1-C_{eff})\sqrt{4KC_{0}F(z)}$$ (12.b)

$$X=aI_{0}H(z)$$ (12.c)

Taylor expending of the ln term in Eq (12.a), we obtain a nonlinear scaling law of I_0_^-0.5^ Bunsen Roscoe law (BRL) [17]. Eq (12) also provide the formula for the crosslink time (T*) which is nonlinearly proportional to X^-0.5^, which may be compared to Eq (6) based on an alternation definition.

For the simple case of Q = p = 0 and D>>1, the S function given by exact formulas (after Wegscheider, 1923) [22]

$$S(z,t)=\sqrt{\left(4KC_{0}/(aI_{0}))(E2/(E2-1)\right)}\text{arctan}(G1)$$ (13.a)

$$G1(z,t)=\sqrt{E2-1}(1-E)/(1+(E2-1)E)$$ (13.b)

where E2 = exp(Az), E = exp(-0.5atI_o_), A = 2.3*ε*_1_C_o_. The steady state solution of Eq (13) is given by when E<0.133, or when the dose E_0_ = tI_o_ > 2.5 (mJ/cm^2^). Which is similar to the condition defined by Eq (11.b). We shall note that there is no exact solution for Eqs (1) or (9) when Q or p is included. Therefore, we solve Eq (1) numerically by finite element method which is justified by comparing the numerical results wit the exact solution, Eq (13) for the special case of Q = p = 0 and D>>1. The roles of Q, p and D in Eq (13) will be fit numerically and presented elsewhere.

## Results and discussions

Unless specified, the following calculated results are based on the measured parameters [17, 20] of *ε*_1_ = 204(%·cm)^-1^ and Q = 13.9 cm^-1^ and the assumed quantum yield *ϕ* = 0.5 and *ε*_2_ = 50 (%·cm)^-1^.

### The efficacy profiles

Fig 2 show the numerically calculated CXL efficacy profiles for UV light intensity at I_0_ = 10 W/cm^2^ and 30 W/cm^2^. The numerical data are consistent with the predictions based on analytic formulas, Eq (11), that for the same UV dose (or energy), higher intensity achieves lower efficacy. Moreover, the efficacy profiles has optical depth (z*) for the transient regime, but not the steady state, where z* is proportional to the dose, or UV exposure time for a given intensity. At the corneal surface (z = 0), the efficacy is independent to the exposure time

Fig 3 shows the profiles of the CXL efficacy for high RF concentration and high power, C_0_ = 0.2% and I_0_ = 30 mW/cm^2.^ for: (A) transient state low doses with exposure time t = (1.0, 1.67, 2.3, 3.3) s and (B) for longer exposure time t = (3,5,7,10) s.

**Fig 4:**
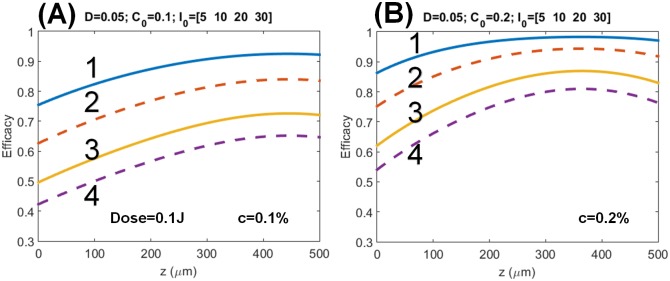
Profiles of the CXL efficacy. Same as Fig 2, but for steady-state and (A) C_0_ = 0.1%, (B) C_0_ = 0.2%, with long exposure time t = (20, 10, 5, 3.33) s, for intensity I_0_ = (5, 10, 20, 30) mW/cm^2^, shown by curves (1, 2, 3, 4), respectively, where all curves has the same dose of 0.1 J/cm^2^.

Fig 4 shows the steady-state CXL efficacy profiles with long exposure time (t) with the same dose of 0.1 J/cm^2^, but for RF concentration C_0_ = 0.1% and C_0_ = 0.2%for intensity I_0_ = (5, 10, 20, 30) mW/cm^2^ and t = (20, 10, 5, 3.33) s. The numerical data are consistent with the predictions based on analytic formulas, Eq (11), that for the same UV dose (or energy), higher intensity achieves lower efficacy, and the efficacy is proportional to the square root of [C_0_/I_o_]. The efficacy steady state value are analytically available from Eqs (9) and (11) with P = 1.

**Fig 5:**
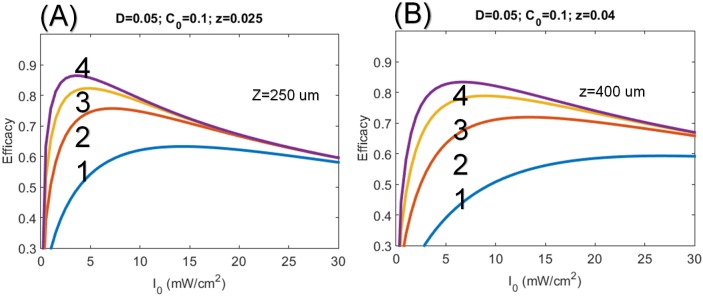
Profiles of the CXL efficacy. CXL efficacy versus UV light intensity for C_0_ = 0.1% and for depth: (A) z = 250 um and (B) 400 um, for exposure time t = (2, 4, 6, 8) s, shown by curves (1, 2, 3, 4), respectively.

Fig 5 shows the optimal intensity of CXL efficacy at depth of 250 and 400 um, where the saturation and optimal features, with optimal intensity (I*) ranging 3 to 12 mW/cm^2^ (for z = 250 um), 3 to 12 mW/cm^2^ (for z = 400 um). These features are also predicted by Eq (11), and they are inverse proportional to the exposure time.

### The nonlinear law

As predicted by Eqs (9) and (11), and our numerical data, the CXL efficacy at transient state (for small dose) is proportional to [t^2^I_0_]^0.5^ and at steady-state, it is a nonlinear increasing function of [C_0_/I_0_] ^0.5^ or [t/E_0_]^0.5^. This nonlinear scaling law is clinically more accurate than that of the linear theory of Bunsen Roscoe law (BRL) [17] and may be used as the new guidance for the protocol of accelerated CXL in relating the exposure time and the UV light intensity. The conventional accelerated CXL protocol based on BRL, therefore, has undervalued the exposure time (t) for higher intensity using the linear scaling of t = [E_0_ /I_0_], rather than t = [E_0_ /I_0_]^0.5^, if based on our nonlinear law. To achieve the same CXL efficacy higher RF concentration requires higher UV light intensity; and for the same dose, higher UV light intensity requires a longer exposure time.

The recent clinical data [23] showed that for the same dose, 9 W/cm^2^ and 10 minutes is more efficient than 30 W/cm^2^ and 3 minutes (based on BRL). This clinical new finding is consistent with our theory (as shown in Figs 2 to 4). However, the theory presented by Kling and Hafezi [23] based on a simplified model same as that of Schumacher [18] predict incorrect efficacy (or stiffness) profiles comparing to our numerical exact solutions. For examples: Fig 1 of Kling and Hafezi [23] did not show the optimal features as that of our Figs 3 and 4; their Fig 5 shows thin cornea has higher efficacy than thick cornea, which is opposite to our predicted feature shown in our Figs 2 to 4 that CXL efficacy always lower at small z (or corneal depth), except at the transient state which shows an optimal z*. The simplified model [18, 23] ignoring the RF depletion, or assuming a constant C(z,t) in our Eqs (1.a) and (1.b), totally eliminate the important competing feature between the UV light intensity and the RF concentration. Therefore their efficacy profiles are incorrect, although Kling and Hafezi [23] claimed that their clinical data (shown by their Fig 4) are consistent with their modeling. We believe that a wrong modeling can still fit to correct clinical data when there is free adjusting parameters in the modeling.

### The demarcation line and crosslink depth

Gatzioufas et al. [24] and Moramarco et al. [25] reported the CXL efficacy and its relation to the stromal demarcation line depth (SDD). The mean SDD were reported at 149 um and 213 um, respectively, in continuous wave (CW) and pulsed light-assisted CXL. Therefore pulsed light-assisted CXL could be more clinically effective than standard CW light. These findings also support the importance of oxygenation for effective CXL, as suggested by Richoz et al. [26].

However, Gatzioufas et al. [24] stated the open debating issues that whether the SDD was a true indicator of CXL efficacy; "the deeper, the better" principle may apply to CXL or other factors interfered with the clinical interpretation of the SDD after CXL. It was reported that the high UV light intensity achieved more superficial the stromal demarcation line [18]. However, there was no proportional decrease in CXL efficacy with increasing UVA light intensity [19].

The above controversial issues may be interpreted by our theory as follow. As shown by Figs 2 and 3, the optimal crosslinking depth (z*) is an increasing function of E_0_, D and *ϕ*, but it is a decreasing function of C_0_. In comparison, the CXL efficacy, Eqs (9) and (11), is an increasing function of C_0_ and the UV exposure time (for a fixed UV intensity, as shown by Fig 3), but it is a decreasing function of UV light intensity (at a fixed dose as shown by Fig 4). As expected, higher concentration results in a stronger absorption of the UV light and reduces the crosslinking depth (z*). Therefore, the CXL efficacy should be characterized by both z* and Ceff, which have opposite trend with respective to the UV light intensity and RF concentration.

Our nonlinear scaling laws demonstrate that, for the same dose, higher UVA light intensity achieves lower Ceff than that of lower intensity. It is known that the elasticity of the anterior corneal stroma is significantly higher than that of the posterior corneal stroma. Therefore, thicker corneas require a larger z* (or longer exposure time) than that of thin corneas to achieve the same CXL efficacy. As suggested by Gatzioufas et al. [24], the minimal (or optimal) amount of dose (energy) and shorter treatment time (using higher intensity) might further improve the CXL safety and reduce its potential complications.

### Corneal thinning effects

It was reported that corneal thinning occurs during intra-operatively during the UV exposure. The thinning effect may be analyzed by the role of the diffusion depth (D) defined in Eq (2.d) as follows. The thinner cornea results a larger effective riboflavin diffusion depth (D) in the stroma and therefore increases the crosslink depth (z*) which can be estimated by Eqs (2) and (10). For example, a typical thinning of 100 um and D increases from 300 um to 400 um, our calculations show a larger z*. However, the currently available clinical data are not sufficient for our modeling to include the actual influence of the thinning effects on the CXL efficacy. Further investigations are required.

Our one-dimensional modeling assumes a uniform distribution of riboflavin across the corneal surface (horizontal direction on the x-y plane), but focuses on the distribution along the depth (z) is included. A two-dimensional model is in progress to include the horizontal features which may be compared to topography data with variation on the x-y plan.

### Maximal efficacy

CXL efficacy may be described by either the measured demarcation line depth or by the increase in stiffness of the crosslinked collagen. Maximal CXL efficacy and crosslink depth would require optimization of the UV light intensity (or dose) for a given riboflavin concentration. As shown in Fig 5, the optimal intensity ranges from 10 to 30 mW/cm^2^ for the maximal CXL efficacy. To improve the CXL efficacy, various techniques have been proposed. These include: pulsed mode operation of the UV light, extra oxygen supply to the corneal surface; and enhancement of the riboflavin diffusion such as diffusion in the de-epithelialized stroma (standard method); diffusion through the epithelium into the stroma (transepithelial method); or direct introduction of riboflavin into the stroma (pocket technique, ring technique, needle technique); and enrichment of riboflavin in the stroma by iontophoresis. In addition to UVA light activated riboflavin, other photosensitizers using blue light (at 430 nm) and green light (at 532 nm) were also proposed.

Detailed photochemical kinetics of CXL was reported showing the roles of oxygen in both type-I and type-II reactions [3, 22]. In addition, combination of supplemental oxygen and pulsing UV exposure providing substantial improvement of CXL efficacy was also reported by Muller et al. (Avedro Inc internal Report). Epi-off technique has been known to be much more efficient than the epi-on which has much less oxygen in the stroma.

We should note that our nonlinear scaling law and the related stromal demarcation line depth require further clinical studies to customize its value under various CXL conditions. These factors include the concentration and diffusion profile of the RF in the stroma, the absorption constant of the photolysis products and the quantum yield of the CXL process, besides the operation modes (CW or pulsed) of the UV light which may influence the oxygen environment in a type-II process.

## Conclusion

To conclude the significance and new findings of this theoretical study of CXL are summarized as follows:

1. The conventional accelerated CXL protocol based on BRL has undervalued the exposure time (t) for higher intensity using the linear scaling of t = [E_0_/I_0_]. In contrast, this study presents, for the first time, the nonlinear scaling law given by t = [E_0_/I_0_]^0.5^ for the CXL efficacy given by Eqs (9) and (11). Our nonlinear theory predicts that longer exposure time than that of BRL is required for UV intensity higher than 3 mW/cm^2^. This new feature is consistent with recent measurements. [7, 18, 19] which did not follow the BRL.
2. The CXL efficacy should be characterized by both optimal crosslink depth (z*) and maximal Ceff, which have opposite trend with respective to the UV light intensity and RF concentration.
3. The efficacy profiles are calculated numerically showing the optimal features and the steady state profiles which are inverse proportional to the UV light intensity (for a give dose, as shown by Figs 3 and 4). Therefore low intensity is always more efficient than the high intensity for the same dose. This new efficacy profiles are different from the profiles derived from a simplified model [20, 23].
4. Depending on the UV exposure time and diffusion depth, the optimal UV intensity ranges from 3 to 30 mW/cm^2^ for maximal CXL efficacy (as shown by Fig 5).
